# Investigating global phase diagrams (GPDs) with reentrant transition behavior

**DOI:** 10.1371/journal.pone.0199459

**Published:** 2018-07-12

**Authors:** Jude Simons Bayor, Baohua Teng, Lingli Wang

**Affiliations:** 1 Department of Applied Physics, Faculty of Applied Sciences, University for Development Studies, Navrongo Campus, Tamale, Ghana; 2 Condensed Matter Physics Department, School of Physical Electronics, University of Electronic Science and Technology of China, Chengdu, People’s Republic of China; Coventry University, UNITED KINGDOM

## Abstract

In this paper we calculate the global phase diagrams with the closed-loop behavior for the phase transition of physical systems by means of the transverse field Ising model with nearest neighbor interaction. The 3D graph plotted by the various physical parameters gives a clear appreciation and qualitative understanding of the reentrant phase behavior of the system. Meanwhile the results show the close correlation between experimental phenomena and our theoretical calculation for the closed-loop behavior for the phase transition of the systems.

## Introduction

Phase transition phenomena occur frequently in the natural world [1] and they are of special interest in many fields of study [2]. Hence the shapes of the various phase diagrams have been obtained theoretically by various models and they have significantly enhanced the understanding of the underlying physical property changes [3–29]. Campi et al [28], reports that the lattice gas is viewed as having similar perspective to that of simple fluids, which exhibits a reversed U-shaped phase diagram with the critical point at the top. Also, some complex fluids (examples being certain micro emulsions) with short range interactions exhibit U-shaped phase diagram with the critical point at the bottom. This is observed in the mixture of water and oil in the presence of a surfactant [28]. In fact some more exotic phase diagrams for complex fluids have been reported [16]. For example a solution of nicotine in water, has a closed-loop shaped phase diagram with double critical points [8]. These reentrant phenomena are also reported in various order-disorder systems, such as the composite structure system of potassium [30], the mixed-spin Ising ferrimagnet system [31], the transverse Ising nanosystem [32], and the solvent-mediated protein system [33]. Recently Bayor et al [34] studied the reentrant phase behavior and obtained closed looped shapes that are coincident with the phenomena exhibited in some proteins, colloids and complex fluid mixtures.

In order to investigate systematically the phase behavior in binary systems and other physical systems, the concept of the global phase diagram (GPD) is usually introduced. Earliest studies in global phase diagrams can be alluded to Konynenburg and Scot [35] in a seminal paper of 1980 where they introduced a classification of phase behavior. Goldstein et al [36] studied rather complex, orientationally specific pair interactions, like those found in real systems by determining the model parameters and thus mapping experiments onto the global phase diagrams. Kolafa et al [37] discovered the azeotropic effect in the complete global phase diagrams for two model binary fluid mixtures described by the one-fluid van der Waals and attractive hard sphere equations of state. GPD not only offers an ideal framework for the theoretical understanding of phase transitions, but also provides the basic relation between phase behavior and underlying intermolecular interactions [37]. Meanwhile the global phase diagram has its origin in the studies of the reappearing phases in some complex systems such as binary fluid mixtures, polar substances, ionic systems, polymer solutions or blends. Of specific interest was how the size and shapes of the closed loops varied with various parameters [38].

In this study we will present the latest calculations of the global phase diagrams by means of the transverse field Ising model with nearest neighbor interaction. Here we will aim at obtaining an agreement between the known experimental phenomena and those from our calculated model and seek to reveal general relationships. The 3D-graph plot of the various physical parameters that we present here, will give a clear appreciation and qualitative understanding of the progressive effect of each parameter change. Also, our GPDs presented, will give the futuristic framework for prediction of the mechanism of the transitions in various systems to guide experimental work that have not been carried out. This is based on the close correlation between present experimental results and our theoretical calculation. It will also afford the possibility to obtain phase diagrams and the critical properties of imaginary or hypothetical physical systems. Once the general phase behavior of condensed matter systems is indispensable and of fundamental interest, an in-depth knowledge by way of our global phase diagrams, will make it easier to translate underlying behavior of systems to offer more complete characterization.

## Materials and methods

### The model

Usually the transverse Ising model can be used to describe the reentrant phase behavior of the phase diagram of complex fluids [20–24], and its Hamiltonian is as follows [20–24,34]

$$H=-\sum_{i}\Omega S_{i}^{x}-\sum_{\langle i,j\rangle }JS_{i}^{z}S_{j}^{z}$$ (1)

Where $s_{i}^{x}$ and $s_{i}^{z}$ are the x- and z-components of a pseudospin-1/2 operator at site i in the lattice, and $\underset{<i,j>}{\sum }$ runs over only nearest-neighboring pairs. Ω is the tunneling interaction parameter and J the exchange interaction parameter.

Using the mean-field approximation, the average occupation number or concentration *ρ* of the site i of the square lattice can be written as [20–24,34]

$$\rho =1/2+\left(\sigma /2\omega_{0}\right)\;\tanh\;(\omega_{0}/2k_{B}T)$$ (2)

where $\omega_{0}^{2}{=\Omega }^{2}{+\sigma }^{2}$ $\sigma =\sum_{i}{J<S}_{i}^{z}>$, and <Siz>=ρ‑12

Here the ensemble average of the pseudo-spin ${<S}_{i}^{z}>$ is the order parameter of the system, and can describe the transition of the system from order given as

$$\langle S_{i}^{z}\rangle \neq 0$$ (3)

to a disordered state represented as

$$\langle S_{i}^{z}\rangle =0$$ (4)

Usually we use it to calculate the concentration ρ and analyze the transition behaviors for complex fluid systems [28].

It is established from experimental research in complex fluids that a temperature increase in systems may result in the increase of interaction parameter [11–12], while for some crystal materials the interaction parameter shows positive relationship with temperature [18–19]. Hence with the concept of effective exchange interaction and the temperature dependence, Campi and Krivine [28] obtained closed-loop shaped phase diagrams with the Ising model, and described the reentrant phase behavior of complex fluids. Additionally we suppose in the transverse Ising model, that the effective exchange and effective transverse field parameters *J* and Ω have simple temperature-dependent relations as in Eqs 5 and 6 [34]:

$$J=J_{0}{\left(\frac{T}{T_{0}}\right)}^{n}$$ (5)

$$\Omega =\Omega_{0}{\left(\frac{T}{T_{0}}\right)}^{m}$$ (6)

where *T*_0_ are arbitrary constants. By this therefore, we can calculate the global phase diagrams involving temperature, concentration, and various interaction parameters for the transverse Ising model with effective temperature-dependent parameters.

## Discussions

### The global phase diagrams

In this section we obtain the global phase diagrams by solving the formulations in section 2. In calculation, the effective parameters *J*_0_, Ω_0_ and *k*_*B*_*T* are reduced by *k*_*B*_*T*_0_, and for simple formalism they are notated still as *J*_0_, Ω_0_ and *t*. In this work we obtain four broad categories of global phase diagrams, show the combined effects of J_0_, Ω_0_, n and m, and describe their topology and phase transitions in details. For each GPD, any vertical “slice” through the section of the global phase diagram represents the phase diagram for a system with particular values of *J*_0_, Ω_0_, m or n strength.

**Fig 1:**
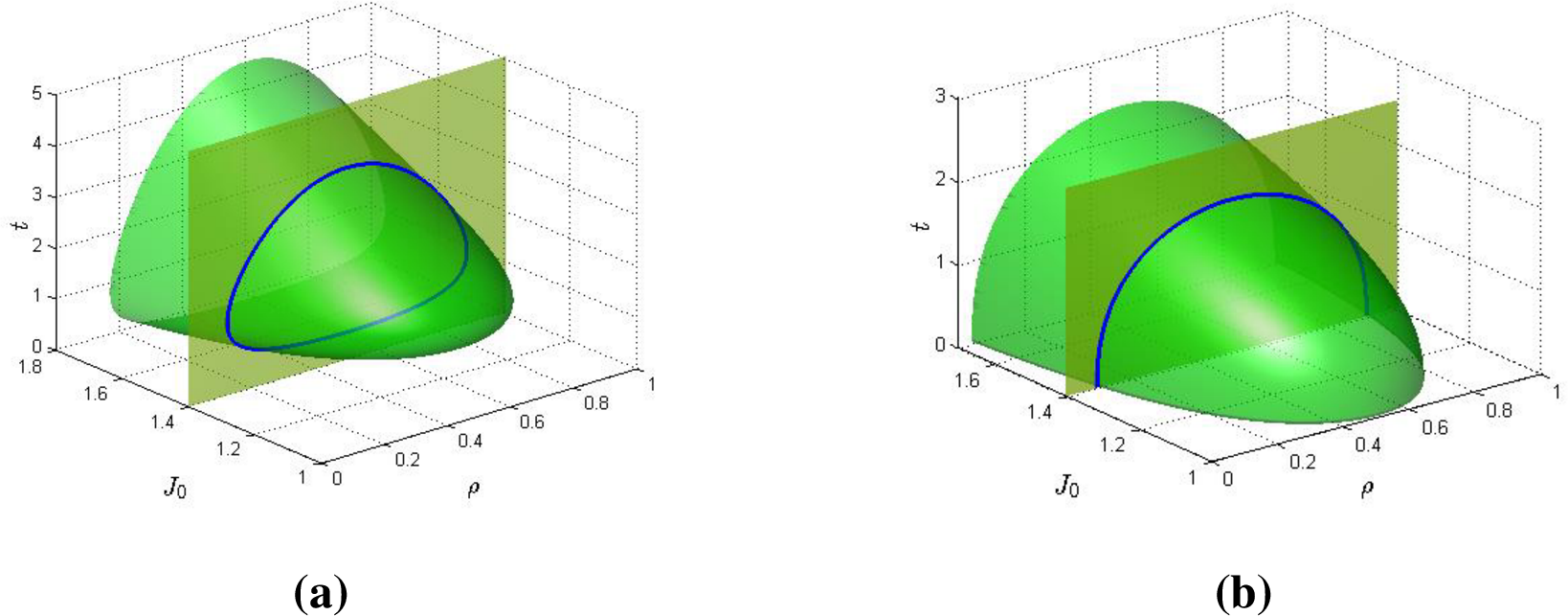
The 3D global phase diagram of the exchange parameter *J*_0_ against temperature *t* and concentration *ρ* for Ω_0_ = 1.10 and for (a) n = 1.5, m = 2.2, and (b) n = 1.0, m = 2.2.

At first category for which the effective field interaction Ω_0_ is fixed, let us show what shape of global phase diagrams can be obtained for the exchange interaction J_0_ in a case where exponent values of m and n are modified. Fig 1(A) shows such a result, in this case starting with large values of m (= 2.2) and n (= 1.5). The global phase diagram has the protuberance of a horn or the section of the nose of a jet plane with the bulging tip oriented towards lower J_0_ value and the open end lies towards higher J_0_ value. Below a value about J_0_ = 1.15, the system is completely disordered under all changes in concentration and temperature. However, at a value of just about 1.15, the system shows a coincident double critical point at lower temperature. At this point the system is thus largely disordered for most temperature ranges. Above J_0_ = 1.15, the egg shaped, closed-loop phase diagram appears with double critical points. An upper critical point corresponds to higher temperatures while its corresponding lower critical point corresponds to lower temperature. As J_0_ increases the closed looped shape increases and locus of points becomes broader. Hence the ordered phase becomes more prominent with increase in J_0_. This means that the system is largely in the ordered state with increase of J_0_. It thus portrays a system with reappearing phases. This phase diagram is coincident with those obtained in our previous work for proteins, colloids and complex fluid mixtures [34]. Typical condensed matter systems that can display this reentrant phase topology include: binary gases, microemulsions, gels, liquid crystals, ferroelectrics granular superconductors, organometallic compounds, etc. [16].

Fig 1(B) shows how the global phase diagram of the effective exchange interaction J_0_ varies with temperature and concentrations of the system and on the sensitivity of its exponent n (as it is lowered to n = 1). The global phase diagram is duck-billed shaped. Here the cross sectional phase diagram shows the standard dome shape. As J_0_ decreases, this dome shape becomes less pitched and the temperature at which order-disorder transitions occur is lowered for any given concentration. For lower J_0_ values, the system is at ordered state at lower temperature and disordered state at higher temperature. At higher J_0_ value the system is at ordered state for a large range of temperature. This phase diagram is reversed U-shaped coincident with some usual ferromagnetic systems. This diagram shows that the transitions are sensitively dependent on exponent n, with the loss of reappearing phase property and the system being in ordered state at low temperatures. These systems are ordered at low temperature and disordered at high temperature as the critical temperature typically increases with exchange interaction J_0_. This situation corresponds to the usual phase diagram of the TIM with effective temperature-independent parameters [34].

**Fig 2:**
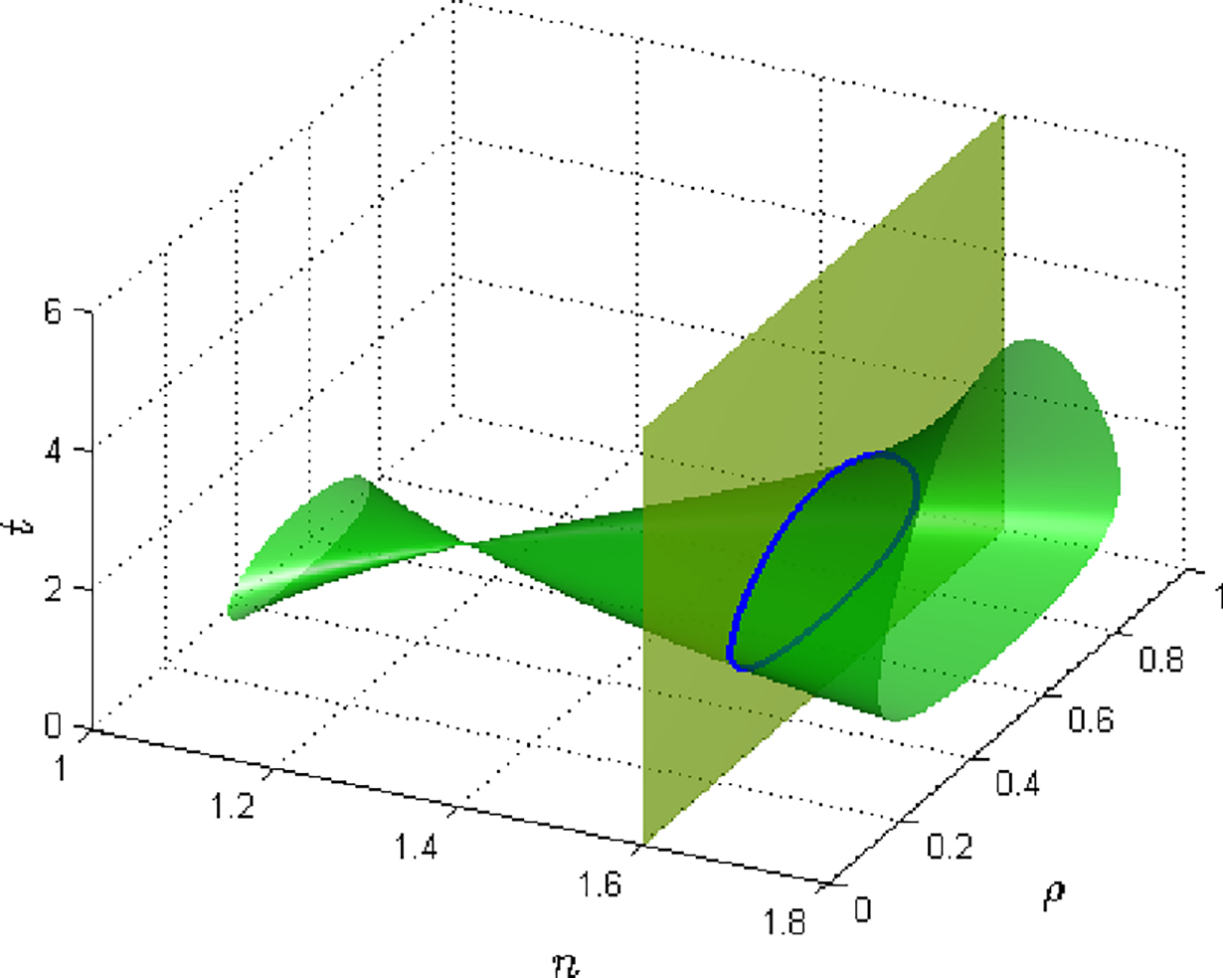
The 3D global phase diagram of the exponent n against temperature *t* and concentration *ρ* for fixed values of *J*_0_ = 1.10, Ω_0_ = 1.11, m = 2.2.

Fig 2 shows the category of global phase diagrams for which we examine the effect of the exponent n on the phase transition of the system and it shows the sensitivity of the effective transverse field Ω_0_. The GPD is vuvuzela shaped or can be viewed laterally as having the shape of an hourglass with two distinct phase ranges. While changing J_0_, Ω_0_, and m, various hourglass shapes are obtainable, some separated at the neck by a gap. The neck (or connection) of the two cone parts as well as the smaller cone are oriented towards lower n while the larger cone is oriented towards higher n. Hence for lower values of n, the system is predominantly at the disordered state at high temperatures and at intermediate temperatures it becomes ordered and for low temperatures it is disordered. The phase diagram is therefore that of a closed looped shape and it shows the characteristics of reappearing phases of the system. The range of concentrations for this phase transition process is very small and thus tapers off and narrows as n increases till at an intermediate point where it again regenerates the closed loop shape. In this part, the phase diagram transcends over a wider range of concentration of the system. Hence at lager values of n, the ordered phase gains some relative prominence in the phase diagram and the reentrant phase behavior with egg-shaped close loop profile is indicative here.

**Fig 3:**
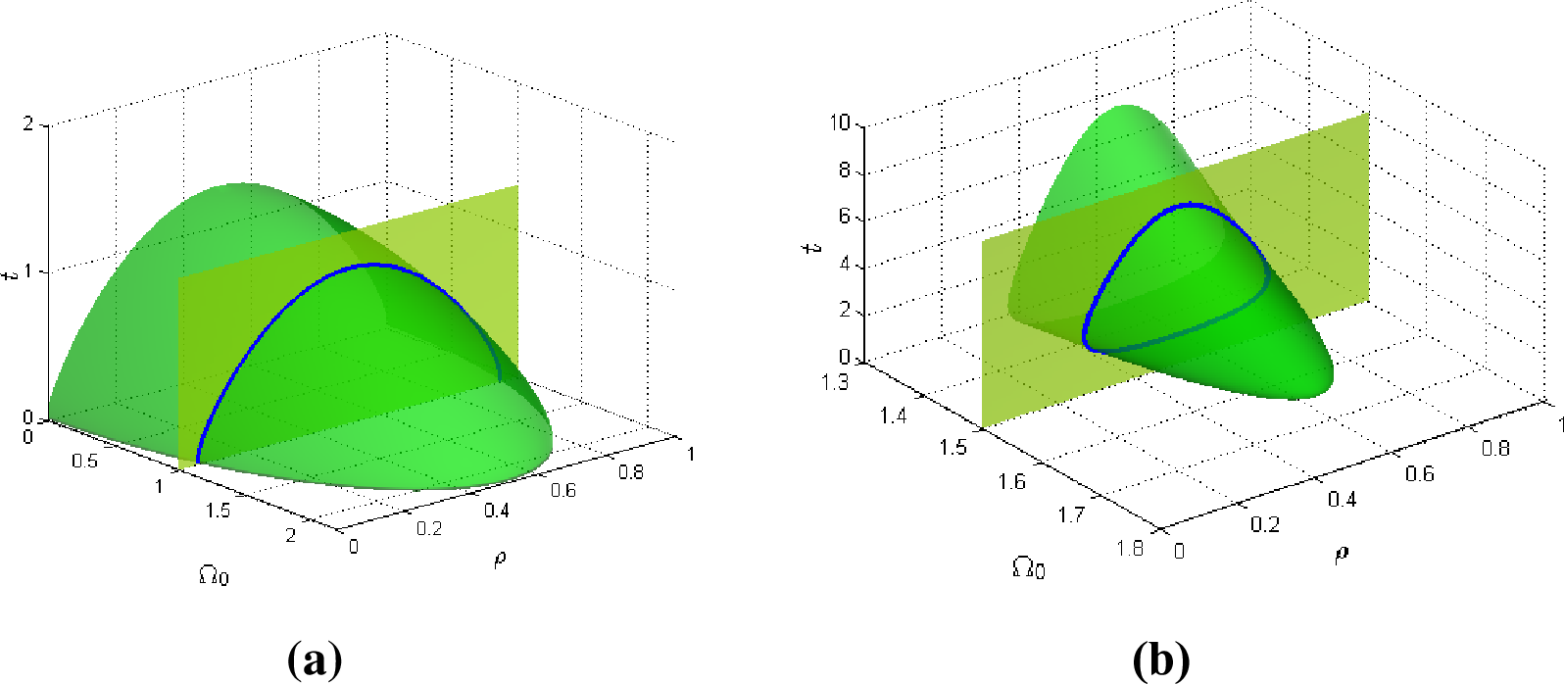
The 3D global phase diagram of the transverse field parameter Ω_0_ against temperature *t* and concentration *ρ* for *J*_0_ = 1.10 and for (a) n = 0.6, m = 0.6, and (b) n = 1.6, m = 1.8.

Fig 3(A) shows the how the effective transverse field Ω_0_ affects the phase diagram of the system. The GPD has the protuberance of the nose of a jet plane. The cross-section phase diagram is dome shaped with the nose oriented towards higher values of Ω_0_ and tapers off. Hence, for high values of Ω_0_, the system is predominantly at the disordered state for a large range of higher temperatures while the ordered state transition occurs at a narrow range of low temperature. As Ω_0_ gets smaller, the ordered state region gets bigger. So with lower Ω_0_, the system is at the ordered state for a progressively wider range of lower temperatures and over wider concentrations as well. This phase diagram is reverse U-shaped and is coincident with usual order-disorder systems. Thus, this parameter has the effect of making the system lose its reappearing phase property.

Fig 3(B) is the GPD showing how the exponents n and m sensitively affect the phase transition diagrams of the system. For the larger values of exponents n and m, the GPD also has the shape of the nose of the jet plane, but the associated phase diagram is closed looped shaped. For high values of Ω_0_ the system is completely at the disordered but as Ω_0_ reduces the ordered phase appears and the phenomenon of reappearing phase predominates. Hence the closed loop phase gets bigger and broader over temperature and concentration ranges as Ω_0_ decreases. As Ω_0_ gets lower, the system is at the ordered state at higher temperatures and largely at disordered state at lower temperatures.

**Fig 4:**
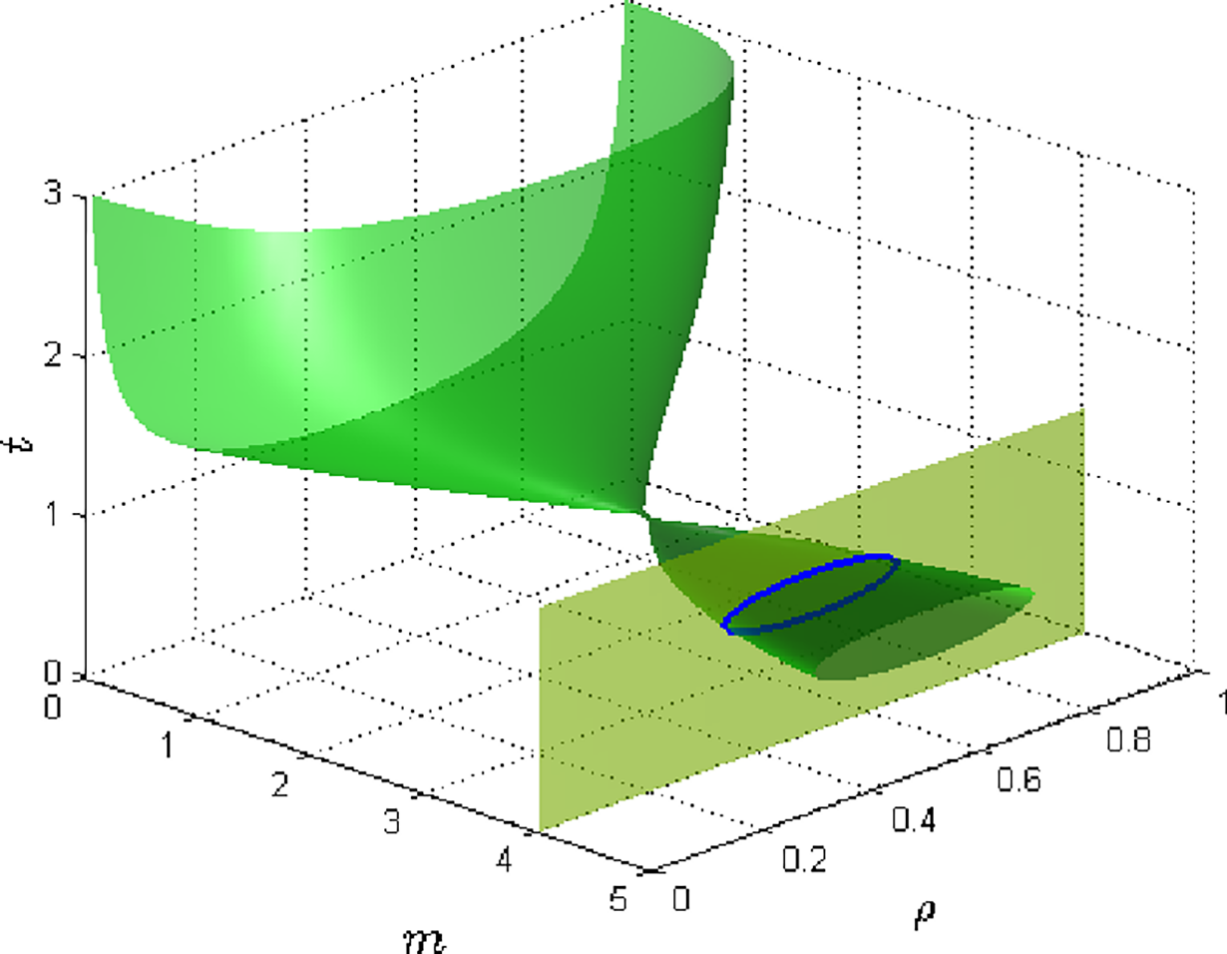
The 3D global phase diagram of the exponent m against temperature *t* and concentration *ρ* for fixed values of *J*_0_ = 1.25,n = 1.6,Ω_0_ = 1.8.

Fig 4 is the category of global phase diagrams showing the effect of the exponent m at varying concentrations and temperatures on the phase transition for systems of given J_0_, n, and Ω_0_. This diagram shows somewhat two part disjointed global phase diagrams. These two distinct phase ranges that can be separated by a gap when J_0_, n, and Ω_0_ are changed. As m increases, the diagram shows two quite distinct transition phase characteristics. For low values of m, the system is predominantly at the ordered state at high temperatures and is at disordered at low temperatures. For low m, phase transition process occurs across the large range of concentration from 0 to 1. The phase diagram here is U-shaped. For intermediate values of m, the system is predominantly at the disordered state but has a coincident critical point where it is in ordered state. Hence the phase diagram contracts from oval O-shaped to a point which is described as the double critical point. It thus shows some semblance of reentrant behavior. For high values of m, the system is at disordered state at high temperatures, and is at ordered state at intermediate temperatures, and finally at disordered state at low temperatures. The phase diagram for this section has closed looped shapes varying from that of regular O-shaped to an oblong O-shape as m increases. These shapes are quite exotic in form. Thus, the phenomenon of reappearing phases for this system is favored when m is high. But the spread of concentration ρ of the system for which the process takes place is smaller for high values of m.

## Conclusions

This paper provides a theoretical model for accurately obtaining a topological phase transition sequel with closed looped phase behaviors, commonly associated with some binary fluids in particular, and for other exotic shapes of phase transition phenomena. By using the transverse Ising model and the supposed exponent dependent relations of the effective interaction parameters on temperature, the 3D global phase diagrams were obtained by the mean-field approximation. Cross sections of these global phase diagrams reveal the snap line phase diagrams for systems at the given concentration. Hence four distinct classifications of systems were encountered. These include the phase diagrams of the egg-shaped closed loops, U-shapes, reversed U-shapes and other exotic topologies commonly observed in experiments.

The numerical calculations indicate that the global phase diagrams depend sensitively and dramatically on the exponents *n* and *m*, and the strength of effective interaction parameters *J*_0_ and Ω_0_. Hence parameter modifications of J_0_ and Ω_0_ change the features of the global phase diagrams between ordered and disordered states exhibiting the reentrant phase behaviors.

Hopefully these results as well as the theoretical techniques can greatly provide useful information for understanding experimental observations.

## Supporting information

S1 Text This is the Matlab code for the Monte Carlo simulation used. (DOCX)
