# Supplementary material for: Investigating global phase diagrams (GPDs) with reentrant transition behavior
Source: PLoS One. 2018 Jul 12;13(7):e0199459. doi: 10.1371/journal.pone.0199459 (PMC6042702; doi:10.1371/journal.pone.0199459)
Supplement: S1 Text — (DOCX) [file pone.0199459.s001.docx]

According to the transcendental equation

$1=[zJ_{0}(T/T_{0})^{n-m}/2\Omega_{0}]\tanh[\Omega_{0}(T/T_{0})^{m}/2k_{B}T]$, (A-1)

the relations between the transition temperature and interaction parameters can be calculated numerically by Monte Carlo simulation and exhibited by the global phase diagram. Here we plot the global phase diagram of exponent n against temperature t and concentration $\rho$ for J_0_ = 1.1, m = 2.2, Ω_0_= 1.1, and z=4. The matlab codes for Monte Carlo simulation are as follows:

J=1.1;m=2.2;Omega=1.1;

k=100;

f = @(n, rho, t) sqrt((4*J.*(t.^n).*(rho-0.5)).^2 + (Omega.*(t.^m)).^2)-2*J.*(t.^n).*tanh(sqrt((4*J.*(t.^n).*(rho-0.5)).^2 + (Omega.*(t.^m)).^2)./(2*t));

n_range=rand(k,1);

rho_range=rand(k,1);

t_range=rand(k*5,1);

n_range=n_range*0.8+1;

t_range=t_range*6;

n_range=sort(n_range);

t_range=sort(t_range);

rho_range=sort(rho_range);

[n, rho, t] = meshgrid(n_range,rho_range, t_range);

v = f(n, rho, t);

isosurface(n, rho, t,v,0);

grid on;
